# Differentially correlated genes in co-expression networks control phenotype transitions

**DOI:** 10.12688/f1000research.9708.1

**Published:** 2016-11-22

**Authors:** Lina D. Thomas, Dariia Vyshenska, Natalia Shulzhenko, Anatoly Yambartsev, Andrey Morgun

**Affiliations:** 1 Instituto de Matemática e Estatística, Universidade de São Paulo, São Paulo, Brazil; 2 College of Pharmacy, Oregon State University, Corvallis, USA; 3 College of Veterinary Medicine, Oregon State University, Corvallis, USA

**Keywords:** co-expression networks, differential co-expression analysis, biological state transition

## Abstract

Background: Co-expression networks are a tool widely used for analysis of “Big Data” in biology that can range from transcriptomes to proteomes, metabolomes and more recently even microbiomes. Several methods were proposed to answer biological questions interrogating these networks. Differential co-expression analysis is a recent approach that measures how gene interactions change when a biological system transitions from one state to another. Although the importance of differentially co-expressed genes to identify dysregulated pathways has been noted, their role in gene regulation is not well studied. Herein we investigated differentially co-expressed genes in a relatively simple mono-causal process (B lymphocyte deficiency) and in a complex multi-causal system (cervical cancer).

Methods: Co-expression networks of B cell deficiency (Control and BcKO) were reconstructed using Pearson correlation coefficient for two *mus musculus* datasets: B10.A strain (12 normal, 12 BcKO) and BALB/c strain (10 normal, 10 BcKO). Co-expression networks of cervical cancer (normal and cancer) were reconstructed using local partial correlation method for five datasets (total of 64 normal, 148 cancer). Differentially correlated pairs were identified along with the location of their genes in BcKO and in cancer networks. Minimum Shortest Path and Bi-partite Betweenness Centrality where statistically evaluated for differentially co-expressed genes in corresponding networks.

Results: We show that in B cell deficiency the differentially co-expressed genes are highly enriched with immunoglobulin genes (causal genes). In cancer we found that differentially co-expressed genes act as “bottlenecks” rather than causal drivers with most flows that come from the key driver genes to the peripheral genes passing through differentially co-expressed genes. Using *in vitro* knockdown experiments for two out of 14 differentially co-expressed genes found in cervical cancer (FGFR2 and CACYBP), we showed that they play regulatory roles in cancer cell growth.

Conclusion: Identifying differentially co-expressed genes in co-expression networks is an important tool in detecting regulatory genes involved in alterations of phenotype.

## Introduction

Recent technological advances have moved the focus of biologists from how to measure biological parameters to how to analyze and interpret tens of thousands of measurements, frequently called omics data. The first solutions for such a problem were limited to hierarchical clustering ^1– 3^ and simple comparisons between classes of data through the identification of differentially expressed genes (DEGs) ^4, 5^. Nowadays, reconstruction and interrogation of biological networks have become a widely used approach to get insights from different types of omics data ^6, 7^.

After establishing co-expression networks for different states of one biological system, differential co-expression analysis investigates their structural changes when a system goes through a state transition. This analysis, first proposed more than a decade ago ^8, 9^, identifies the pairs of genes that have their interaction changed during such transition. Several later publications have suggested different algorithms and statistics to determine differential gene co-expression ^10– 27^. Fewer studies, however, attempted to evaluate the biological significance of these changes ^18, 21^. Also, to the best of our knowledge, there have been no studies that would investigate how this approach performs depending on the type and complexity of the biological system analyzed.

Commonly, a state transition of a biological system is related to perturbation of a set of genes, which propagates through network interactions and affects other genes. Thus, there is a possibility that differentially co-expressed (DC) genes (directly or indirectly) contribute to the propagation of perturbations. In order to investigate the role of DC genes in a state transition of a biological system, we considered two biological processes ^28, 29^ previously analyzed by our group. The first one (B cell deficiency in mice) is a homogenous, one-causal-factor process, while the second one (cervical cancer) represents a heterogeneous multi-causal system.

In this work, a co-expression network is an undirected graph, where the set of nodes consists of a set of DEGs, and a pair of nodes is connected if there is a significant correlation between them. Differential co-expression analysis is done by identifying the pairs of genes that suffer significant changes in correlation between two states. Throughout this paper such pairs are called differentially correlated pairs (DCPs) and the genes forming these pairs are considered DC genes.

## Results

### B cell deficiency

We started by analyzing the B cell knockout (BcKO) data ^28^, which represents a relatively simple experimental model with only one causal factor (B lymphocytes) and homogenous subject groups since this experiment was performed in highly inbred strains of mice.

**Figure 1.**
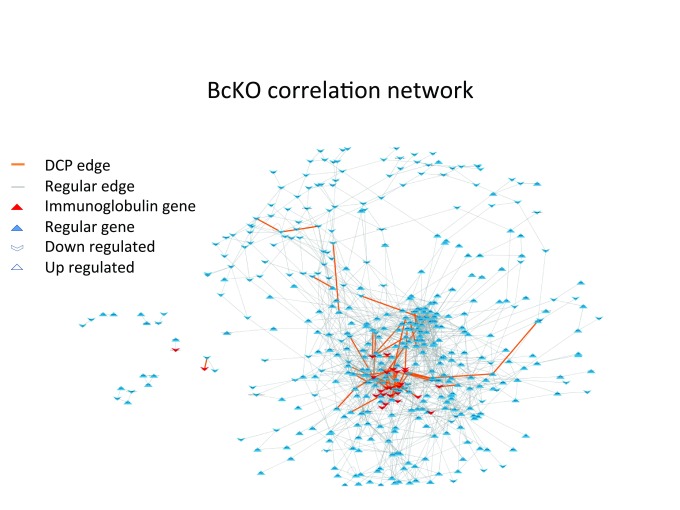
Co-expression networks for BcKO data. The nodes are composed by DEGs and the edges represent significant correlations between nodes. The causal genes (immunoglobulin genes) and the DCP edges are concentrated in the high connectivity region with several causal genes forming DCPs.

In order to select the nodes to reconstruct the co-expression networks (BcKO and Control) we compared gene expression in jejunum between BcKO and control mice and found 509 DEGs ( Dataset 1). Next, the edges for each network were determined using significantly correlated pairs of DEGs ( Figure 1). To identify DCPs we used the method introduced in ^21^ which compares correlations in the BcKO group and in the Control group. Eighty DCPs were found ( Dataset 2), of which 56 represent correlation gains (edges which were not present in Control network but showed up in BcKO) and 24 represent losses.

Differentially expressed genes from BcKO study Contains p-values, ratios of expression means, combined Fisher’s p-value, fdr, direction of regulation, whether it is Ig gene and whether it is DC gene. Click here for additional data file. Copyright: © 2016 Thomas LD et al. 2016 Data associated with the article are available under the terms of the Creative Commons Zero "No rights reserved" data waiver (CC0 1.0 Public domain dedication).

Differentially correlated pairs from BcKO study Contains information such as “change direction” (whether each pair gained or lost correlation/edge), “sign of local partial correlation” in BcKO data and control data, “regulation” (whether each gene of each pair is up- or down-regulated in BcKO), “number of Ig genes” in each pair. Click here for additional data file. Copyright: © 2016 Thomas LD et al. 2016 Data associated with the article are available under the terms of the Creative Commons Zero "No rights reserved" data waiver (CC0 1.0 Public domain dedication).

**Figure 2.**
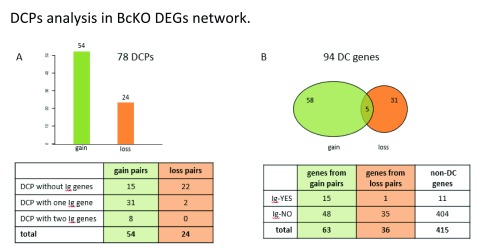
**A**) 78 Differentially Correlated Pairs (DCPs) were found, of which 54 represent correlation gains (edges which were not present in Control network but showed up in BcKO) and 24 represent correlation losses. The table stratifies the set of pairs representing correlation gains and losses according to the amount of Ig genes (0, 1 or 2) present in a pair. Note that 39 out of 54 of correlation gain DCPs are formed by at least one Ig gene while only 2 out of 22 correlation losses have at least one Ig gene. **B**) The 78 DCPs are formed by a total of 94 Differentially Co-expressed genes (DC genes). 58 DC genes participate only in correlation gain DCPs, 31 only in correlation loss DCPs and 5 of them participate in both correlation gain and loss DCPs. The results show enrichment for Ig genes among DC genes in correlation gain: 24% (15 out of 63 (=58+5)) of DC genes are Ig genes vs 2.7% (11 out of 415) of other DEGs are Ig genes (p value < 0.001). Meanwhile no enrichment was observed for correlation loss as a result of B cell deficiency: 3% (1 out of 36 (=31+5)) of DC genes are Ig genes vs 2.7% (11 out of 415) of other DEGs are Ig genes.

Now we investigate whether network structural changes, herein represented by DCPs, are related to actual causes of global change in gene expression. In the previous study ^28^, it was shown that intestinal gene expression alterations in BcKO mice are mostly dependent on the ability of B lymphocytes to produce antibodies. Therefore, we analyzed the presence of immunoglobulin coding genes (Ig genes, see Dataset 3) among differentially expressed genes (26 Ig genes among 509 DEGs) in DCPs. We observed that 72% (39 out of 54) of correlation gain DCPs are formed by at least one Ig gene, ( Figure 2A). Moreover, we found strong enrichment for Ig genes among DC genes in correlation gain (24% (15 out of 63) of DC genes are Ig genes vs 2.7% (11 out of 415) of other DEGs are Ig genes), while no enrichment was observed for correlation lost as a result of B cell deficiency ( Figure 2B). Thus, these results support the idea that differentially expressed genes that acquire correlations during transition from one biological state to another have a high chance to play causal roles in such transition.

Causal genes from BcKO study Contains the Ig genes considered causal along with annotation and whether they are considered DC genes or not. Click here for additional data file. Copyright: © 2016 Thomas LD et al. 2016 Data associated with the article are available under the terms of the Creative Commons Zero "No rights reserved" data waiver (CC0 1.0 Public domain dedication).

### Cervical cancer

***Analysis of gene expression data.*** In order to study differentially co-expressed genes in a more complex biological model we turned to cancer. It is well known that cancers of the same clinically/morphological type can be very different on molecular levels. One of the most studied causes for such diversity is the different sets of chromosomal aberrations and mutations harbored by tumors otherwise defined as the same cancer. In previous study ^29^, we have found 36 cervical cancer driver genes located in multiple chromosomal aberrations ( Dataset 4). Thus we decided to use cervical cancer data from 29 for investigation of the role of DCPs in complex biological processes due to its heterogeneity and previously acquired knowledge of essential causal genes.

Causal genes from cervical cancer study Contains the chromosomal aberration genes considered causal along with annotation and whether they are considered DC genes or not. Click here for additional data file. Copyright: © 2016 Thomas LD et al. 2016 Data associated with the article are available under the terms of the Creative Commons Zero "No rights reserved" data waiver (CC0 1.0 Public domain dedication).

**Figure 3.**
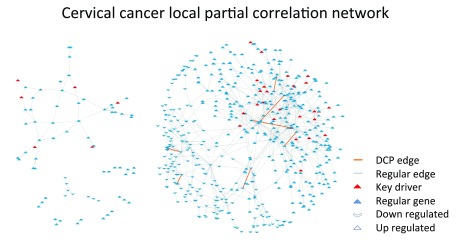
Co-expression networks for cervical cancer data. The nodes are composed by DEGs and the edges represent significant local partial correlation between nodes. A few causal genes (key drivers) and DCP edges are located in the high connectivity region, but scattered throughout the network. Only one key driver is amongst the genes in DCPs.

We used the DEGs between tumor and normal tissue as the nodes of the co-expression networks. Since the number of samples (five datasets, 148 tumor samples and 67 normal samples) was larger than in BcKO study (two datasets, 22 paired samples), we used the partial correlation coefficient as a measure of co-expression ( Figure 3). The potential advantage of using partial correlation is that it aims to infer edges that are a result of direct regulatory relations ^6^. Partial correlations were calculated through the Local Partial Correlation (LCP) method ^30^ ( Material and Methods).

In this study seven DCPs composed of 14 DC genes were found. Interestingly, all DCPs were differential correlations gained in tumors ( Table 1). Only one of the 36 key drivers (CEP70) was identified among the 14 DC genes. Accordingly, no enrichment of key driver genes among DC genes was detected in this analysis.

**Table 1.** DCPs – cancer (* key drivers).

Gene symbol 1	Gene symbol 2	Change direction	Sign of local partial correlation in tumor	Regulation 1	Regulation 2
ANP32E	CACYBP	Gained edge	> 0	UP	UP
CENPN	DHFR	Gained edge	> 0	UP	UP
C10orf68	FGFR2	Gained edge	> 0	DN	DN
AK2	HNRNPR	Gained edge	> 0	UP	UP
CEP70*	SEPHS1	Gained edge	> 0	UP	UP
NIPAL2	TRPM3	Gained edge	> 0	DN	DN
They stem ARHGEF12	ZSCAN18	Gained edge	> 0	DN	DN

Even though we observed that DCPs are not necessarily formed by key drivers, it is known from literature that most of the DC genes found play regulatory roles in other types of cancer ^31– 48^. Thus we hypothesized that DCPs are located downstream of key drivers and can be responsible for changes of regulatory chain events coming from the key drivers and spreading throughout the network. In order to verify this hypothesis, we investigated how close DC genes are to key drivers and whether their “signal flow” ^49^ in the tumor co-expression network is stronger than that of the other genes. In order to verify this hypothesis we investigated two network measures: Minimum Shortest Path and Bi-partite Betweenness Centrality.

**Figure 4.**
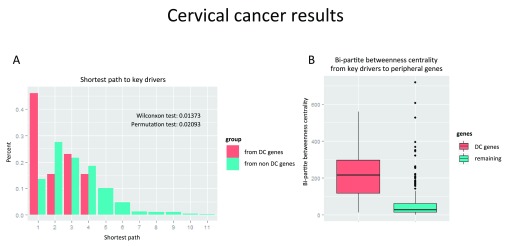
Topological properties of Differentially Correlated Genes (DCGs). **A**) Barplot of the shortest path to the causal genes and originated in either the genes in DCPs (in orange) or the non DCP genes (in blue). The distribution in orange is concentrated in lower values. **B**) Boxplot comparing the values of Bipartite Betweenness Centrality of the genes in DCPs (in orange) and the non-DCP genes (in blue). The boxplot on the left is concentrated in higher values.

First we compared the shortest paths from key driver genes to DC genes and to all other DEGs in the network. We found that DC genes are located statistically closer than the rest of genes in the network to key drivers ( Figure 4A, Wilcoxon test < 0.014 and Permutation test < 0.021). Then we used Bi-partite Betweenness Centrality ^6^ as a measure of the signal flow from key drivers to peripheral genes (genes with only one edge) ^6^. We evaluated this measure for DC genes and remaining DEGs and observed that DC genes had much higher values than other genes in the network. Figure 4B illustrates a comparison of boxplots of bi-partite betweenness centrality between these two groups concerning DCPs and the rest (non DCPs, non-key drivers, non-peripheral). We can observe that the bi-partite betweenness centralities of DCPs are concentrated in higher values than the rest. Mann-Whitney test gave us a p-value of 7.868 X 10 ^-5^, which gives us evidence that the distribution of Bi-Partite Betweenness Centrality in DCP genes is higher. For more details see Figure S2. Thus, altogether these results suggest that DC genes might be “bottlenecks”, that is, required to transmit a signal from key drivers to other genes in the network, therefore, supplement the hypothesis of a regulatory role of DC genes ( Figure S1).

***Knockdown experiments.*** In addition, data from other cancers provide indirect support for the idea of regulatory role of DC genes in cervical cancer ^31– 48^. However, since a role of these DC genes in carcinogenesis was not as straightforward as for immunoglobulin genes in B cell deficiency, we decided to perform experimental tests. Among the DC genes found for cervical cancer, there were seven up-regulated and seven down-regulated in cancer. Therefore, for validation experiments we chose one down-regulated (FGFR2) and one up-regulated (CACYBP) gene that have not been previously studied in cervical cancer for regulatory properties, but have a potential connection with cell death or proliferation based on their Gene Ontology annotations. In order to test if FGFR2 and CACYBP play critical regulatory roles in cancer pathogenesis, we evaluated the effect on *in vitro* knockdown of these genes on cell proliferation in a cervical carcinoma cell line.

**Figure 5.**
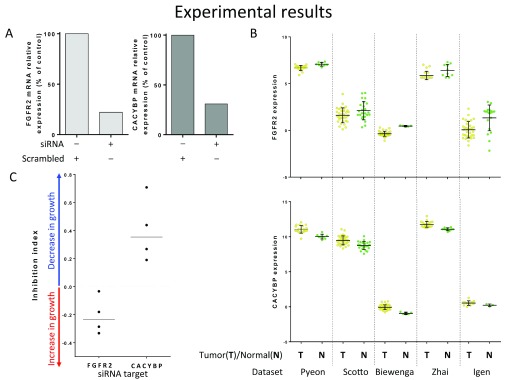
Experimental evaluation of DCGs in cervical cancer. **A**) Efficacy of FGFR2 and CACYBP siRNA knockdown. qRT-PCR with primers for GAPDH as the internal control was used to determine expression and efficacy of FGFR2 and CACYBP specific siRNA knockdown in endothelial cells (ME180). ME180 cells were harvested 72 h after transfection with vehicle (Lipofectamine) and either scrambled control or targeting siRNA. **B**) Gene expression of FGFR2 and CACYBP (mean +/- standard deviation) for tumor and normal samples from five datasets used in the analysis. Since FGFR2 was found down-regulated in tumor tissue, its potential regulatory role would be as a tumor suppressor. However, CACYBP is up-regulated, thus CACYBP should function as an oncogene promoting cell proliferation. **C**) Evaluation of cell proliferation inhibition using xCelligence System. Proliferation data (cell index) was obtained at 72 h after transfection with Lipofectamine and either scrambled control or targeting siRNA. Inhibition index was calculated (two step normalization of cell index): inhibition index > 0 – cells transfected with targeting siRNA showed decrease in proliferation; < 0 – showed increase in proliferation; = 0 – no difference from control was found. One sided T test for mean (< 0 for FGFR2 and > 0 for CACYBP) was applied and returned statistically significant p-values for both of them (0.0258 for FGFR2 and 0.01978 for CACYBP).

First, we tested two cervical cancer cell lines (Hela and ME180) and found that only ME180 had detectable expression levels of both genes. In order to perform these tests, we evaluated siRNAs and observed that they were able to knock down expression of both genes by at least 70% ( Figure 5A). CACYBP is up-regulated in tumor tissue, as compared to normal tissue ( Figure 5B). Consequently, if CACYBP has regulatory potential, as predicted by our analysis, it should function as an oncogene promoting cell proliferation. Therefore, the knockdown of this gene should result in a decrease of cell growth/survival. Since FGFR2 was found down-regulated in cervical carcinomas ( Figure 5B) its potential regulatory role would be as a tumor suppressor. Therefore, the knockdown of this gene is expected to increase cell growth. The subsequent analysis of cell proliferation confirmed our predictions for both genes: knockdown of CACYBP led to a decrease of cell growth, while knockdown of FGFR2 induced higher cell proliferation ( Figure 5C). Thus, these results provide additional support to our *in silico* prediction that DC genes may play a regulatory role in cell proliferation related to tumor growth.

Cytoscape Edges and Nodes tables from network in Figure 1 The datasets are sufficient to reproduce Figure 2. Click here for additional data file. Copyright: © 2016 Thomas LD et al. 2016 Data associated with the article are available under the terms of the Creative Commons Zero "No rights reserved" data waiver (CC0 1.0 Public domain dedication).

Cytoscape Edges and Nodes tables from network in Figure 3 The datasets are sufficient to reproduce Figure 4. Click here for additional data file. Copyright: © 2016 Thomas LD et al. 2016 Data associated with the article are available under the terms of the Creative Commons Zero "No rights reserved" data waiver (CC0 1.0 Public domain dedication).

Raw data for Figure 5A,C Raw data for Figure 5A:
qRT PCR siRNA test.
Instrument Type: steponeplus
Passive Reference: ROX
Analysis Type: Singleplex
Endogenous Control: GAPDH
RQ Min/Max Confidence Level: 95.0
Reference Sample: A
Raw data for Figure 5C:
Three xCellingence experiments. Click here for additional data file. Copyright: © 2016 Thomas LD et al. 2016 Data associated with the article are available under the terms of the Creative Commons Zero "No rights reserved" data waiver (CC0 1.0 Public domain dedication).

## Discussion

In the current study, the differential co-expression analysis ^21^ was applied to two relatively well-investigated biological systems ^28, 29^ in order to evaluate the potential importance of genes found using differential correlation analyses. Overall, the obtained results support the idea that DC genes play a regulatory role. While in B cell deficiency DCPs were found highly enriched with immunoglobulin genes (i.e. causal genes for alterations in the gut) we did not observe enrichment for key driver genes in cervical cancers. Rather, DCPs of cervical cancer seem to be located downstream of causal genes. Indeed, those DCPs have been found closer to key regulators than other genes in the network, actually representing “bottlenecks” for communication between driver genes previously published in 29 and the rest of the network ( Figure 4). Furthermore, some differentially co-expressed genes in cervical cancer have been previously implicated in processes such as metastasis, oncogenic autophagy and apoptosis. For example, CACYBP has been shown to promote colorectal cancer metastasis ^31^, TRPM3 was observed to play a role in oncogenic autophagy in clear cell renal cell carcinoma ^32, 33^, and AK2 was reported to activate apoptotic pathway ^34^. Several genes are investigated for prognostic value for cancers such as myeloma ^35^, lymphoma ^36^, breast ^37– 41^ and gastrointestinal ^42, 43^ cancers. At least two genes were previously proposed as targets for anti-cancer agents: DHFR ^44^ and FGFR2 ^45^. Moreover, CACYBP and ZSCAN18 were also reported as putative tumor suppressor genes in renal cell carcinoma ^30, 46, 47^. In addition, we have tested two DC genes and confirmed their regulatory role (FGFR2 as a tumor suppressor and CACYBP as a potential oncogene in cervical cancer) by manipulating their expression *in vitro*. Altogether, published observations and our experimental validation for these two genes support the idea that DC genes revealed in the current study play a regulatory role and can be candidate targets for cervical cancer treatment.

Interestingly, while in the model of B cell deficiency, the DC genes are highly enriched with causal regulatory genes, there was only one key driver in cervical cancer (CEP70), despite the DC genes in this system still seeming to play a regulatory role overall. Such a difference is potentially related to the fact that the mouse system studied in 28 is highly homogeneous (inbred mice) with only one cause of alterations (i.e. absence of B lymphocytes). Cervical cancer, however, is a heterogeneous system with different chromosomal aberrations and consequently turned-on expression of different driver genes in different patients. Therefore, we can speculate that differential correlations point to regulatory genes that are shared by majority of samples. This hypothesis warrants further investigation, especially considering that DCPs could represent common therapeutic targets for tumors that originated as a result of different genomic or epi-genomic events.

In conclusion, this study provided additional evidence for the previously suggested idea ^8– 27^ that genes presenting alterations in correlation patterns between different phenotypes (i.e. states of biological system) play a critical regulatory role in transitions from one state to another. Furthermore, although our results do not allow for full generalization, they indicate that gain and not loss of correlations connects critical genes involved in transitions to new phenotypes. However, further studies are required to understand how changes in correlation patterns can point to genes with critical capacity to guide a biological system into certain state/phenotype.

## Material and methods

### Preparation of microarray data

***BcKO.*** All microarray data were analyzed using BRB Array-Tools developed by the Biometric Research Branch of the National Cancer Institute under the direction of R. Simon ( http://linus.nci.nih.gov/BRB-ArrayTools.html). Array data were filtered to limit analysis to probes with greater than 50% of samples showing spot intensities of >10 and spot sizes >10 pixels, and a median normalization was applied.

***Cervical cancer.*** Same as in cervical cancer ^29^. The data were analyzed using BRB Array-Tools using the original normalization used in three studies ^50– 52^ and median normalization over entire the array for the fourth study ^53^. For all studies, we only considered genes found in at least 70% of arrays.

### Filtering and meta-analysis of microarray data

In every analysis (DEGs, DCPs and networks), filter of direction (same sign of correspondent parameter – difference of mean, difference of correlation, correlation and partial correlation) was required in a fixed number of datasets (2 out of 2 in BcKO and 3 out of 5 in cervical cancer). Then meta-analysis was done through Fisher combined probability test ^54^. Next, the pairs with false discovery rate (fdr) ^55^ lower than a threshold are chosen. At last, only the pairs that pass PUC ^56^ are considered correlated and therefore represent edges in the network.

### Analysis of microarray data

***BcKO.*** DEGs between groups of samples were identified by random variance paired t-test p-value lower than 5% with adjustment for multiple hypotheses by setting the fdr below 10% in BRB Array-Tools **.** Co-expression networks (BcKO and Control) were inferred through Pearson correlation with p-value < 20% and fdr adjustment below 2.5%. DCPs were calculated for pairs that were initially correlated (p-value < 20%) in at least one state. Then differences of Pearson correlation were tested following ^21^ with a p-value below 10% and fdr < 2%. At last only the DCPs that showed up in one of the networks were selected.

***Cervical cancer.*** DEGs were retrieved from a cervical cancer paper ^29^. Correlation networks and DCPs followed the same procedure and in BcKO but with different p-values (correlation p-value < 10% with fdr < 10 ^-8^ and difference of correlation p-value < 10% with fdr < 0.25%). Partial correlation was computed using local partial correlation method ^30^. The initial significance was p-value lower than 40% and then fdr < 5%.

For more details about the thresholds used, see Table S3 and Table S4.

### Local partial correlation network

Two aspects of cervical cancer data motivated us to use local partial correlation for this system. First of all, we have more samples throughout five datasets (see Supplementary Table S1 and Supplementary Table S2) which allows us to have more confidence in our results and second we already know that tumors in general present heterogeneous causal factors. The partial correlation approach gives us the alternative to only consider edges that represent direct regulatory relations.

**Figure 6.**
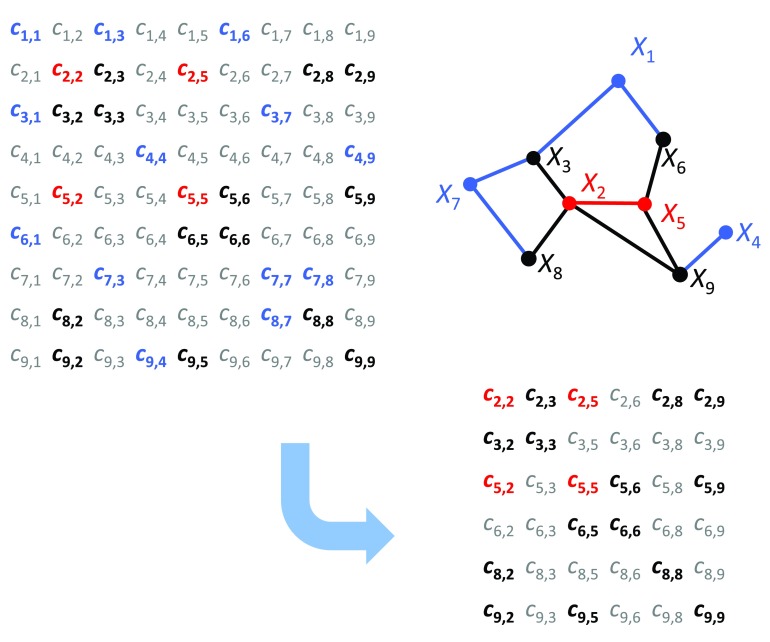
Local partial correlation scheme: we calculate the LPC for pair *X*_2_, *X*_5_, (red nodes/edge). The neighborhood of this pair is the set of nodes *X*_3_, *X*_6_, *X*_8_, *X*_9_ (black nodes/edges). *X*_1_, *X*_4_, *X*_7_ (blue nodes) are significantly correlated with the black nodes (blue edges), but not with the red nodes. Thus the inverse method is applied exclusively to the correlation sub-matrix formed only by the genes *X*_2_, *X*_5_, *X*_3_, *X*_6_, *X*_8_, *X*_9_. In correlation matrices the gray entries are statistically non-significant empirical correlations.

In this paper we used the new approach developed in 30 called local partial correlation. This approach was elaborated specially for cases when there are more variables than samples, which happens regularly in genetics and is a serious problem in classical statistics. First we calculate the correlation network. Then for each significantly correlated pair the inverse method is applied exclusively to the correlation sub-matrix formed only by the closest neighbors of the pair along with the genes forming the pair, Figure 6. If the number of closest neighbors is still higher than the number of samples n, then we decreasingly rank the correlations of the neighbors to either genes in the pair and select the first n/2 neighbors. For each sub- matrix, we only keep the partial correlation value regarding the pair that formed that sub- matrix and then calculate its p-value also based on the sub- matrix. R script for calculation is available in Supplementary Material.

Partial correlations were estimated only for the significant (Pearson) correlations in co-expression network. Thus the same definition of DCPs (by Pearson correlation) can still represent structural changes as long as it remains present in one of the two networks.

Figure 3 illustrates the local partial correlation network for cervical cancer using only tumor data. It has 578 connected nodes and 824 edges.

### Minimum shortest path

**Figure 7.**
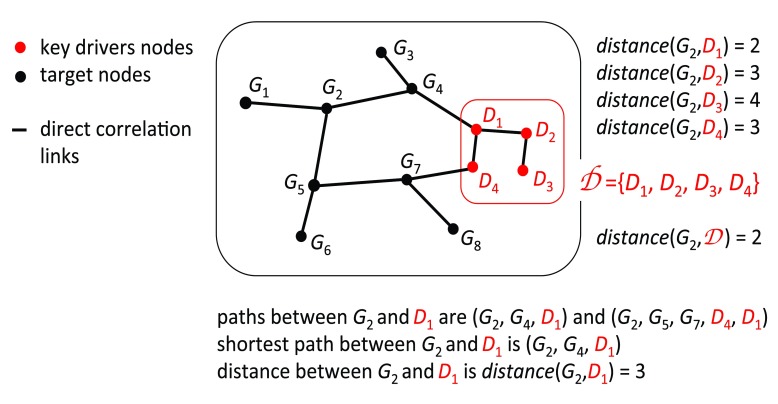
In this example we show how to calculate the distance (length of shortest path) between the gene *G*_2_ and group of genes *D*_1_, *D*_2_, *D*_3_, *D*_4_ (nodes in red).

The shortest path is a method that calculates distances between 2 nodes in a network. It consists of the minimum number of edges connecting 2 nodes. In this case we want to know the minimum number of edges connecting one node, either DCP gene or not, to a group of nodes: the key drivers Figure 7. For each gene we calculate the shortest path to all key drivers and get the minimum value. Then we compare the minimum shortest path to key drivers coming from DCP genes and the remaining genes. Figure 4A shows that the minimum shortest path to key drivers tend to be smaller when originated in DCP genes.

### Bi-partite betweenness centrality

**Figure 8.**
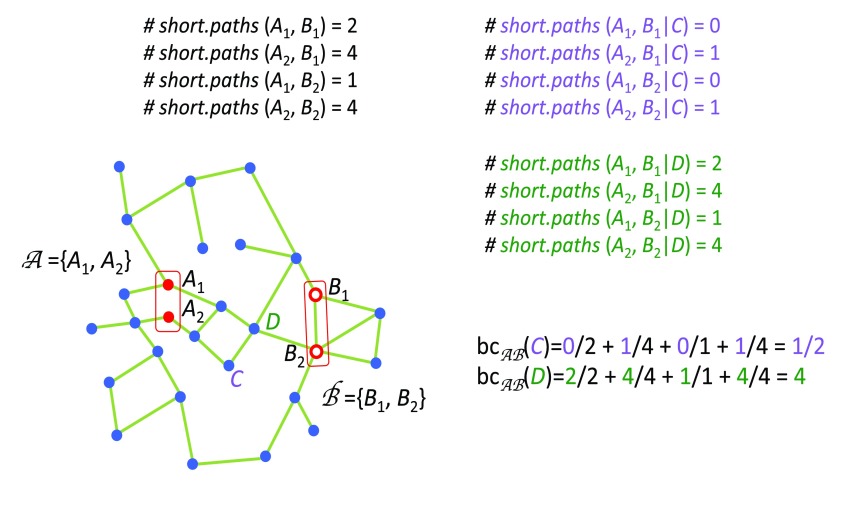
Here we explain how to calculate bi-partite betweenness centrality **(bc)** between groups *A* and *B*. Note that the node *D* has bigger bc because all shortest paths connecting nodes in group *A* to nodes in group *B* pass through the node *D*.

Betweenness Centrality measures the node’s centrality in a network by counting the number of shortest paths from all vertices to all other vertices that pass through that node. A gene with high betweenness centrality has a great influence on the transfer of signal through the network Figure 8.

However we are interested in the signal passing from key drivers throughout the network. For this reason we decided to apply the measure previously developed by our lab ^6^ called Bi-partite Betweenness Centrality. It measures the amount of shortest path going from all genes in one group of vertices to all genes in a different group of vertices. In our case, the groups of genes are the key drivers and the peripheral genes (genes connected to only one edge).

## Experimental design

### FGFR2 and CACYBP knockdown experiment

ME180 cells were transfected with FGFR2-, CACYBP-specific siRNA or control siRNA using Lipofectamine RNAiMAX Transfection Reagent. Cell growth rate during 72h after siRNA transfection was measured using xCelligence system as described below.

***Evaluation of siRNA efficacy in knocking down the gene targets.*** ME180 cell line was obtained from Dr. Pulivarthi H. Rao. It was cultured in RPMI medium with 10% FBS and 1% Penicillin-Streptomycin added. The cells were seeded at density 4000 cells per well in 96 F-bottom plates (seeding procedure was done according to ATCC protocol for ME180 cell line) and with cell culture media 200 ul per well. 24 hours after seeding, cells were transfected with one of the three siRNA, see Table 2.

**Table 2.** Suppliers.

Target	Supplier	Supplier ID
FGFR2	ThermoFisher	s5173
CACYBP	ThermoFisher	s25819
Non-targeting siRNA	Dharmacon	D-001810-01-05

Before transfection, 100 uL of media was taken from each well. Transfection procedure was done according to Lipofectamine RNAiMAX Reagent protocol (Protocol Pub. No. MAN0007825 Rev. 1.0). 3pM of siRNA per well and Lipofectamine 0.6 uL per well were delivered in 20uL. 80 uL of fresh cell culture media was added to each well.

Cells were collected 72 h after transfection using Lysis buffer from RNeasy Mini Kit (QIAGEN). RNA extraction was done using RNeasy Mini Kit (QIAGEN) according to the manufacturer’s protocol (no Dnase treatment step was done). Concentrations of RNA measured with Qubit RNA BR Assay Kit. cDNA was done using Bio-Rad iScript cDNA Kit according to the manufacturer’s protocol.

Quantitative Real-Time PCR was done for the samples using QuantiFast SYBR Green PCR Kit and GAPDH as a control gene. Primers for the targets you can see in the Table 3.

**Table 3.** Primers and Targets.

Target	Forward/ Reverse	Primer sequence (5' -> 3')
FGFR2	Forward	AACAGTTTCGGCTGAGTCCAG
FGFR2	Reverse	GCCCAGTGTCAGCTTATCTCTT
CACYBP	Forward	CTCTGTGGAAGGCAGTTCAAA
CACYBP	Reverse	TCAGGTAATCCCACCTTGTGTT
GAPDH	Forward	GGAGCGAGATCCCTCCAAAAT
GAPDH	Reverse	GGCTGTTGTCATACTTCTCATGG

qRT PCR set up: sample was heated to 95°C, followed by 40 cycles of 95°C for 10 sec and 60°C for 30 sec.

***Evaluation of cell growth after knock down of gene targets.*** CACYBP is up-regulated in tumor tissue, as compared to normal tissue ( Figure 5B). Consequently, if CACYBP has regulatory potential, as predicted by our analysis, it should function as an oncogene promoting cell proliferation. Therefore, the knockdown of this gene should result in a decrease of cell growth/survival. Since FGFR2 was found down-regulated in cervical carcinomas ( Figure 5B) its potential regulatory role would be as a tumor suppressor. Therefore, the knockdown of this gene is expected to increase cell growth.

Cell growth was evaluated using xCelligence system (The RTCA DP Instrument) using manufacturer’s protocol. ME180 was cultured in RPMI media with 10% FBS and 1% Penicillin-Streptomycin added. The cells were seeded at density 4000 cells per well (E-Plate 16) in 200 uL of cell culture media.

24 hours after seeding, the experiment was paused for transfecton. Before transfection, 100 uL of media was taken from each well. Transfection procedure was done according to Lipofectamine RNAiMAX Reagent protocol (Protocol Pub. No. MAN0007825 Rev. 1.0). 3pM of siRNA per well and Lipofectamine 0.6 uL per well were delivered in 20uL; 80 uL of fresh cell culture media was added to each well. Plate was placed back in the slot and cell growth was evaluated for another 72 h.

***Cell index normalization.*** To evaluate cell growth rate cell index was transformed into Inhibition index in two steps:

1. Cell indexes for all wells were exported to the excel file. For each treatment (including non-targeting siRNA transfected wells) we extracted cell index average for all wells at 20 h after seeding ( *Cell Index Before Treatment*) and at 96 h after seeding ( *Cell Index After Treatment*). To normalize cell index to initial cell number differences for each of the treatments we used the following formula:
  $$\text{After/Before}\;\text{Treatment}\;\text{Normalized}\;\text{Cell}\;\text{Index}\;\text{(A/B}\;\text{Index)}=\frac{\mathrm{CellindexAfterTreatment}}{\mathrm{CellindexBeforeTreatment}}$$
2. In next step we normalized each treatment with targeting siRNA to treatment with non-targeting siRNA. For this purpose in each experiment A/B Index from treatment (siRNA targeting either FGFR2 or CACYBP) was normalized to A/B Index from control treatment using the following formula:
  $$\text{Inhibition}\;\text{Index}=\frac{\mathrm{Control}\;\mathrm{A/B}\;\mathrm{Index–Treatment}\;\mathrm{A/B}\;\mathrm{Index}}{\mathrm{Control}\;\mathrm{A/B}\;\mathrm{Index}}$$

Final evaluation of growth was done according to the value of Inhibition Index:

>0 – there is a decrease in growth;

0 – no difference between treated with targeting and treated with non-targeting siRNA;

<0 – there is a growth after treating with targeting siRNA.

## Data availability

The data referenced by this article are under copyright with the following copyright statement: Copyright: © 2016 Thomas LD et al.

Data associated with the article are available under the terms of the Creative Commons Zero "No rights reserved" data waiver (CC0 1.0 Public domain dedication). 

**BcKO:** Gene expression files containing array data from 28 are available under the GSE23934 superseries in the Gene Expression Omnibus (GEO) data repository. We worked with two groups of samples: B10.A littermates and BALB/C (Table S1).

**Cervical cancer:** We have used the same datasets as in previous study ^29^ available at GEO: GSE7410 ^50^, GSE6791 ^51^, GSE7803 ^52^, GSE9750 ^53^, GSE26342 ^29^ ( Table S21).

*F1000Research*: Dataset 1. Differentially expressed genes from BcKO study, 10.5256/f1000research.9708.d142100 ^57^

*F1000Research*: Dataset 2. Differentially correlated pairs from BcKO study, 10.5256/f1000research.9708.d142099 ^58^

*F1000Research*: Dataset 3. Causal genes from BcKO study, 10.5256/f1000research.9708.d142097 ^59^

*F1000Research*: Dataset 4. Causal genes from cervical cancer study, 10.5256/f1000research.9708.d142098 ^60^

*F1000Research*: Dataset 5. Cytoscape Edges and Nodes tables from network in Figure 1, 10.5256/f1000research.9708.d142101 ^61^

*F1000Research*: Dataset 6. Cytoscape Edges and Nodes tables from network in Figure 3, 10.5256/f1000research.9708.d142102 ^62^

*F1000Research*: Dataset 7. Raw data for Figure 5A,C, 10.5256/f1000research.9708.d142103 ^63^

## Supplementary material

Extra details, figures and tables

## References

[1] Kaufman L Rousseeuw PJ: Finding Groups in Data: An Introduction to Cluster Analysis. (1 ed.). New York: John Wiley. ISBN 0-471-87876-6,1990 Reference Source

[2] Press WH Teukolsky SA Vetterling WT: Section 16.4. Hierarchical Clustering by Phylogenetic Trees. *Numerical Recipes: The Art of Scientific Computing (3rd ed.).*New York: Cambridge University Press, ISBN 978-0-521-88068-8,2007 Reference Source

[3] Hastie T Tibshirani R Friedman J: 14.3.12 Hierarchical clustering. *The Elements of Statistical Learning*(PDF) (2nd ed.). New York: Springer. 520–528, ISBN 0-387-84857-6,2009.

[4] Dudoit S Yang YH Callow MJ: Statistical methods for identifying differentially expressed genes in replicated cDNA microarray experiments. *Stat Sin.* 2002;12(1):111–139. Reference Source

[5] Reiner A Yekutieli D Benjamini Y: Identifying differentially expressed genes using false discovery rate controlling procedures. *Bioinformatics.* 2003;19(3):368–375. 10.1093/bioinformatics/btf877 12584122

[6] Dong X Yambartsev A Ramsey SA: Reverse enGENEering of Regulatory Networks from Big Data: A Roadmap for Biologists. *Bioinform Biol Insights.* 2015;9:61–74. 10.4137/BBI.S12467 25983554 PMC4415676

[7] Morgun A Dzutsev A Dong X: Uncovering effects of antibiotics on the host and microbiota using transkingdom gene networks. *Gut.* 2015;64(11):1732–43. 10.1136/gutjnl-2014-308820 25614621 PMC5166700

[8] Kostka D Spang R: Finding disease specific alterations in the co-expression of genes. *Bioinformatics.* 2004;20(Suppl 1):i194–9. 10.1093/bioinformatics/bth909 15262799

[9] Xiao Y Frisina R Gordon A: Multivariate search for differentially expressed gene combinations. *BMC bioinformatics.* 2004;5(1):164. 10.1186/1471-2105-5-164 15507138 PMC529250

[10] Shin E Yoon Y Ahn J: TC-VGC: a tumor classification system using variations in genes’ correlation. *Comput Methods Programs Biomed.* 2011;104(3):e87–e101. 10.1016/j.cmpb.2011.03.002 21531474

[11] Needham M Hu R Dwarkadas S: Hierarchical parallelization of gene differential association analysis. *BMC Bioinformatics.* 2011;12:374. 10.1186/1471-2105-12-374 21936916 PMC3248234

[12] Amar D Safer H Shamir R: Dissection of regulatory networks that are altered in disease via differential co-expression. *PLoS Comput Biol.* 2013;9(3):e1002955. 10.1371/journal.pcbi.1002955 23505361 PMC3591264

[13] de la Fuente A: From ‘differential expression’ to ‘differential networking’- identification of dysfunctional regulatory networks in diseases. *Trends Genet.* 2010;26(7):326–333. 10.1016/j.tig.2010.05.001 20570387

[14] Lai Y Wu B Chen L: A statistical method for identifying differential gene-gene co-expression patterns. *Bioinformatics.* 2004;20(17):3146–3155. 10.1093/bioinformatics/bth379 15231528

[15] Li KC: Genome-wide coexpression dynamics: theory and application. *Proc Natl Acad Sci U S A.* 2002;99(26):16875–16880. 10.1073/pnas.252466999 12486219 PMC139237

[16] Dettling M Gabrielson E Parmigiani G: Searching for differentially expressed gene combinations. *Genome Biol.* 2005;6(10):R88. 10.1186/gb-2005-6-10-r88 16207359 PMC1257471

[17] Watson M: CoXpress: differential co-expression in gene expression data. *BMC Bioinformatics.* 2006;7:509. 10.1186/1471-2105-7-509 17116249 PMC1660556

[18] Mani KM Lefebvre C Wang K: A systems biology approach to prediction of oncogenes and molecular perturbation targets in B-cell lymphomas. *Mol Syst Biol.* 2008;4:169. 10.1038/msb.2008.2 18277385 PMC2267731

[19] Hu R Qiu X Glazko G: Detecting intergene correlation changes in microarray analysis: a new approach to gene selection. *BMC Bioinformatics.* 2009;10:20. 10.1186/1471-2105-10-20 19146700 PMC2657217

[20] Cho SB Kim J Kim JH: Identifying set-wise differential co-expression in gene expression microarray data. *BMC Bioinformatics.* 2009;10:109. 10.1186/1471-2105-10-109 19371436 PMC2679020

[21] Skinner J Kotliarov Y Varma S: Construct and Compare Gene Coexpression Networks with DAPfinder and DAPview. *BMC Bioinformatics.* 2011;12:286. 10.1186/1471-2105-12-286 21756334 PMC3149583

[22] Dawson JA Ye S Kendziorski C: R/EBcoexpress: an empirical Bayesian framework for discovering differential co-expression. *Bioinformatics.* 2012;28(14):1939–40. 10.1093/bioinformatics/bts268 22595207 PMC3492001

[23] Fukushima A: DiffCorr: an R package to analyze and visualize differential correlations in biological networks. *Gene.* 2013;518(1):209–214. 10.1016/j.gene.2012.11.028 23246976

[24] Jacob L Neuvial P Dudoit S: Package ‘DEGraph’.2012 Reference Source

[25] Choi JK Yu U Yoo OJ: Differential coexpression analysis using microarray data and its application to human cancer. *Bioinformatics.* 2005;21(24):4348–4355. 10.1093/bioinformatics/bti722 16234317

[26] Pronk TE van Someren EP Stierum RH: Unraveling toxicological mechanisms and predicting toxicity classes with gene dysregulation networks. *J Appl Toxicol.* 2013;33(12):1407–1415. 10.1002/jat.2800 22886929

[27] Cho DY Kim YA Przytycka TM: Chapter 5: Network biology approach to complex diseases. *PLoS Comput Biol.* 2012;8(12):e1002820. 10.1371/journal.pcbi.1002820 23300411 PMC3531284

[28] Shulzhenko N Morgun A Hsiao W: Crosstalk between B lymphocytes, microbiota and the intestinal epithelium governs immunity versus metabolism in the gut. *Nat Med.* 2011;17(12):1585–1593. 10.1038/nm.2505 22101768 PMC3902046

[29] Mine KL Shulzhenko N Yambartsev A: Gene network reconstruction reveals cell cycle and antiviral genes as major drivers of cervical cancer. *Nat Commun.* 2013;4: 1806. 10.1038/ncomms2693 23651994 PMC4237593

[30] Thomas LD Fossaluza V Yambartsev A: Building complex networks through classical and bayesian statistics - a comparison. In *XI Brazilian Meeting on Bayesian Statistics* AIP Conf. Proc.2012;1490:323–331. 10.1063/1.4759617

[31] Ghosh D Li Z Tan XF: iTRAQ Based Quantitative Proteomics Approach Validated the Role of Calcyclin Binding Protein (CacyBP) in Promoting Colorectal Cancer Metastasis. *Mol Cell Proteomics.* 2013;12(7):1865–1880. 10.1074/mcp.M112.023085 23543800 PMC3708172

[32] Cost NG Czyzyk-Krzeska MF: Regulation of autophagy by two products of one gene: TRPM3 and miR-204. *Mol Cell Oncol.* 2015;2(4):e1002712, in press. 10.1080/23723556.2014.1002712 27308495 PMC4905337

[33] Hall DP Cost NG Hegde S: TRPM3 and miR-204 establish a regulatory circuit that controls oncogenic autophagy in clear cell renal cell carcinoma. *Cancer cell.* 2014;26(5):738–753. 10.1016/j.ccell.2014.09.015 25517751 PMC4269832

[34] Lee HJ Pyo JO Oh Y: AK2 activates a novel apoptotic pathway through formation of a complex with FADD and caspase-10. *Nat Cell Biol.* 2007;9(11):1303–1310. 10.1038/ncb1650 17952061

[35] Walker BA Leone PE Chiecchio L: A compendium of myeloma-associated chromosomal copy number abnormalities and their prognostic value. *Blood.* 2010;116(15):e56–e65. 10.1182/blood-2010-04-279596 20616218

[36] Björck E Ek S Landgren O: High expression of cyclin B1 predicts a favorable outcome in patients with follicular lymphoma. *Blood.* 2005;105(7):2908–2915. 10.1182/blood-2004-07-2721 15576476

[37] Landemaine T Jackson A Bellahcène A: A six-gene signature predicting breast cancer lung metastasis. *Cancer Res.* 2008;68(15):6092–6099. 10.1158/0008-5472.CAN-08-0436 18676831

[38] Nie F Yu XL Wang XG: Down-regulation of CacyBP is associated with poor prognosis and the effects on COX-2 expression in breast cancer. *Int J Oncol.* 2010;37(5):1261–1269. 10.3892/ijo_00000777 20878073

[39] Horlings HM Lai C Nuyten DS: Integration of DNA copy number alterations and prognostic gene expression signatures in breast cancer patients. *Clin Cancer Res.* 2010;16(2):651–663. 10.1158/1078-0432.CCR-09-0709 20068109

[40] Hunter DJ Kraft P Jacobs KB: A genome-wide association study identifies alleles in *FGFR2* associated with risk of sporadic postmenopausal breast cancer. *Nat Genet.* 2007;39(7):870–874. 10.1038/ng2075 17529973 PMC3493132

[41] Katoh M: Cancer genomics and genetics of FGFR2 (Review). *Int J Oncol.* 2008;33(2):233–237. 10.3892/ijo_00000001 18636142

[42] Jang JH Shin KH Park JG: Mutations in *fibroblast growth factor receptor 2* and *fibroblast growth factor receptor 3* genes associated with human gastric and colorectal cancers. *Cancer Res.* 2001;61(9):3541–3543. 11325814

[43] Vedeld HM Andresen K Eilertsen IA: The novel colorectal cancer biomarkers *CDO1, ZSCAN18* and *ZNF331* are frequently methylated across gastrointestinal cancers. *Int J Cancer.* 2015;136(4):844–853. 10.1002/ijc.29039 24948044 PMC4277335

[44] Berman EM Werbel LM: The renewed potential for folate antagonists in contemporary cancer chemotherapy. *J Med Chem.* 1991;34(2):479–485. 10.1021/jm00106a001 1995868

[45] Kwak Y Cho H Hur W: Antitumor effects and mechanisms of AZD4547 on FGFR2-deregulated endometrial cancer cells. *Mol Cancer Ther.* 2015;14(10):2292–2302. 10.1158/1535-7163.MCT-15-0032 26294741

[46] Ning X Sun S Hong L: Calcyclin-binding protein inhibits proliferation, tumorigenicity, and invasion of gastric cancer. *Mol Cancer Res.* 2007;5(12):1254–1262. 10.1158/1541-7786.MCR-06-0426 18171983

[47] Sun S Ning X Liu J: Overexpressed CacyBP/SIP leads to the suppression of growth in renal cell carcinoma. *Biochem Biophys Res Commun.* 2007;356(4):864–871. 10.1016/j.bbrc.2007.03.080 17400182

[48] Morris MR Ricketts CJ Gentle D: Genome-wide methylation analysis identifies epigenetically inactivated candidate tumour suppressor genes in renal cell carcinoma. *Oncogene.* 2011;30(12):1390–1401. 10.1038/onc.2010.525 21132003

[49] Weng G Bhalla US Iyengar R: Complexity in biological signaling systems. *Science.* 1999;284(5411):92–96. 10.1126/science.284.5411.92 10102825 PMC3773983

[50] Biewenga P Buist MR Moerland PD: Gene expression in early stage cervical cancer. *Gynecol Oncol.* 2008;108(3):520–526. 10.1016/j.ygyno.2007.11.024 18191186

[51] Pyeon D Newton MA Lambert PF: Fundamental differences in cell cycle deregulation in human papillomavirus-positive and human papillomavirus-negative head/neck and cervical cancers. *Cancer Res.* 2007;67(10):4605–4619. 10.1158/0008-5472.CAN-06-3619 17510386 PMC2858285

[52] Zhai Y Kuick R Nan B: Gene expression analysis of preinvasive and invasive cervical squamous cell carcinomas identifies *HOXC10* as a key mediator of invasion. *Cancer Res.* 2007;67(21):10163–10172. 10.1158/0008-5472.CAN-07-2056 17974957

[53] Scotto L Narayan G Nandula SV: Identification of copy number gain and overexpressed genes on chromosome arm 20q by an integrative genomic approach in cervical cancer: potential role in progression. *Genes Chromosomes Cancer.* 2008;47(9):755–765. 10.1002/gcc.20577 18506748

[54] Fisher RA: Statistical Methods for Research Workers. Oliver and Boyd (Edinburgh);1925 Reference Source

[55] Benjamini Y Hochberg Y: Controlling the false discovery rate: a practical and powerful approach to multiple testing. *J R Stat Soc B.* 1995;57(1):289–300. Reference Source

[56] Yambartsev A Perlin M Kovchegov Y: Unexpected links reflect the noise in networks. *Biol Direct.* 2016;11(1):52. 10.1186/s13062-016-0155-0 27737689 PMC5480421

[57] Thomas LD Vyshenska D Shulzhenko N: Dataset 1 in: Differentially correlated genes in co-expression networks control phenotype transitions. *F1000Research.* 2016 Data Source 10.12688/f1000research.9708.1 PMC5247791 28163897

[58] Thomas LD Vyshenska D Shulzhenko N: Dataset 2 in: Differentially correlated genes in co-expression networks control phenotype transitions. *F1000Research.* 2016 Data Source 10.12688/f1000research.9708.1 PMC5247791 28163897

[59] Thomas LD Vyshenska D Shulzhenko N: Dataset 3 in: Differentially correlated genes in co-expression networks control phenotype transitions. *F1000Research.* 2016 Data Source 10.12688/f1000research.9708.1 PMC5247791 28163897

[60] Thomas LD Vyshenska D Shulzhenko N: Dataset 4 in: Differentially correlated genes in co-expression networks control phenotype transitions. *F1000Research.* 2016 Data Source 10.12688/f1000research.9708.1 PMC5247791 28163897

[61] Thomas LD Vyshenska D Shulzhenko N: Dataset 5 in: Differentially correlated genes in co-expression networks control phenotype transitions. *F1000Research.* 2016 Data Source 10.12688/f1000research.9708.1 PMC5247791 28163897

[62] Thomas LD Vyshenska D Shulzhenko N: Dataset 6 in: Differentially correlated genes in co-expression networks control phenotype transitions. *F1000Research.* 2016 Data Source 10.12688/f1000research.9708.1 PMC5247791 28163897

[63] Thomas LD Vyshenska D Shulzhenko N: Dataset 7 in: Differentially correlated genes in co-expression networks control phenotype transitions. *F1000Research.* 2016 Data Source 10.12688/f1000research.9708.1 PMC5247791 28163897

