# Supplementary material for: Differentially correlated genes in co-expression networks control phenotype transitions
Source: F1000Res. 2016 Nov 22;5:2740. [Version 1] doi: 10.12688/f1000research.9708.1 (PMC5247791; doi:10.12688/f1000research.9708.1)
Supplement: Supplementary file 8 [file f1000research-5-10464-s0007.tgz › 99b4e4c8-4a30-45ce-b7f7-748edac05142.docx]

**Supplementary Information**

**Title: Differentially correlated genes in co-expression networks control phenotype transitions**

Lina D. Thomas, Dariia Vyshenska, Natalia Shulzhenko, Anatoly Yambartsev, Andriy Morgun

Correspondence to:

A. Morgun: [andriy.morgun@oregonstate.edu](mailto:andriy.morgun@oregonstate.edu)

A. Yambartsev: [yambar@ime.usp.br](mailto:yambar@ime.usp.br)

**Content:**

Supplementary Methods & References

**Supplementary Methods**

Datasets and their causal genes

We decided to work with data in Shulzhenko et al [28] and Mine et all [29] because of the information provided by their research about the genes triggering the changes in gene interactions in both systems (BcKO and cervical cancer) and, consequently, in phenotype. In this work, these genes are also referred to as causal genes. The first paper studied the effects in gene-gene interactions of B cell Knockout mice while the second studied gene expression DEGs network of cervical cancer and identified DEGs located in the regions of frequent chromosomal aberrations. Since we used some of their results, such as causal genes and DEGs, we used the same datasets and normalization as in these papers.

**Bcell Knockout (BcKO)**

Gene Expression Omnibus (GEO) data repository information can be found in Supplementary Table S1 along with reference, strain background, sample size, platform and number of transcripts.

Data processing: After subtraction of local background, signal intensity values were filtered to remove those lower than 10. We only considered genes with gene symbol and present in at least 70% of arrays. Data from GEO was already processed: log 2 transformation and median normalization.

Causal genes: Immunoglobulin genes expression is limited to B lymphocytes As expected in the gut of B cell deficient mice these genes have much lower expression comparing to control mice as well [28]. Since we have experimentally demonstrated in our previous study [28] that it gene expression phenotype observed in Bcell deficient intestines is predominantly dependent on ability of B cells to produce antibodies, we considered immunoglobulin genes as causal genes in analysis and they can be found in Dataset 1.

**Cervical Cancer**

Mine et al [28] searched PubMed at the NCBI database (http://www.ncbi.nlm.nih.gov/pubmed/) for studies of microarray in cervical cancer (published until 03/2009) and selected four studies [50 -53] that: (i) had publicly available microarray data, (ii) used tumor and normal clinical samples, (iii) used oligonucleotide arrays and (iv) had sample size in each class $\geq$ 5 (Supplementary Table S2). Besides publicly available data, they also analyzed gene expression from cervical cancer biopsies and normal adjacent tissue samples.

Gene Expression Omnibus (GEO) data repository information can be found in Supplementary Table S2 along with reference, sample size, platform and number of transcripts.

Data processing: Just like in [28], for all studies, we only considered genes found in at least 70% of arrays. Data were already normalized in the data repository, except for data from [53], to which we applied median normalization, exactly like in [29].

Causal Genes: It was proven in [29] that there is a strong association between chromosomal aberrations and DEGs which shows that most of the DEGs located in the regions of frequent chromosomal aberrations are causal genes. We used the causal genes provided in [29] (Dataset 2).

Supplementary Table S1: Datasets included in the meta-analysis of gene expression microarray data for Bcell Knockout.

| **Reference** | **Accesion Number** | **Strain** | **# normal mice** | **# BcKO mice** | **Array Platform** | **Approximate number of transcripts** |
| --- | --- | --- | --- | --- | --- | --- |
| Shulzhenko et al, 2011 | GSE23573 | B10.A littermates | 12 | 12 | NIAID Mmca -- Mouse | 38K |
| Shulzhenko et al, 2011 | GSE23573 | BALB/c | 10 | 10 | NIAID Mmca -- Mouse | 38K |

Supplementary Table S2: Datasets included in the meta-analysis of gene expression microarray data for cervical cancer

| **Reference** | **Accesion Number** | **# normal tissue samples** | **# tumor tissue samples** | **Array Platform** | **Approximate**  **number of transcripts** |
| --- | --- | --- | --- | --- | --- |
| Mine et al, 2013 | GSE26342 | 20 | 40 | In house, NIAID, NIH | 14K |
| Biewenga et al, 2008 | GSE7410 | 5 | 35 | Agilent-012391 G4112A | 41K |
| Scotto et al, 2008 | GSE9750 | 21 | 32 | Affymetrix HG-U133A | 39K |
| Pyeon et al, 2007 | GSE6791 | 8 | 20 | Affymetrix HG-U133_Plus_2 | 47K |
| Zhai et al, 2007 | GSE7803 | 10 | 21 | Affymetrix HG-U133A | 39K |

Differentially Expressed Genes (DEGs)

**B cell Knockout**

Since in [28] genes expressed in B cells were excluded from the analysis, we decided to identify DEGs considering all genes. It was done separately for each strain using the Excel add-in BRB – Array Tools Version 4.4.1 Stable developed by Dr. Richard Simon & BRB-ArrayTools Development Team in DCTD: Biometric Research Program at NIH National Cancer Institute. The univariate test used to test if there was expression mean change between states was the Paired T-test (with random variance model).

From this point forward, the DEGs analysis was done in the statistical software R version 3.1.2. The two tables were merged based on probe IDs. Then only genes present in both studies were considered. Finally, we did the meta-analysis by calculating Fisher p-value [54], also known as Fisher’s method or Fisher’s combined probability test, and then applied Benjamini Hochberg false discovery rate (FDR) [55] on Fisher p-value. Only genes with FDR lower than 0.1 were considered differentially expressed genes.

In order to keep genes that are individually alike we applied the following filters:

- Same direction of regulation in all studies (either up or down regulated in all studies)
- Individual t-test p-value < 0.05

We ended up with 584 DEGs (509 removing Gene symbol duplicates).

Table with BcKO DEGs in Dataset 4

**Cervical Cancer**

The 1268 DEGs analyzed in this work from cervical cancer data have been previously discovered in [29] by comparing gene expression from tumor and normal samples.

Network Reconstruction

**Correlation**

The procedure used to build a correlation network was the same as in [6]. It basically consists of computing the correlation (here we worked with Pearson correlation) for all pairs of DEGs for each dataset separately as well as states. Then, for each state, only pairs that present the same direction of correlation (sign) in all studies and correlation p-value lower than a threshold are kept. These two filters only take place on studies where both genes in a pair are present. Then we perform meta-analysis by combining the correlation p-values through Fisher’s method. False discovery rate (Benjamini Hochberg FDR) is then applied on Fisher p-value. Next, the pairs with FDR lower than a threshold are chosen. At last, only the pairs that pass PUC [56] are considered correlated and therefore represent edges in the network. Figure 1 illustrates the BcKO network with 433 connected nodes and 1583 edges.

The filters and thresholds used to build the networks for BcKO and cervical cancer can be found in Table S3 and Table S4 respectively. In each system, the values are the same for both states.

Supplementary Table S3: All filters for all calculations on BcKO data

|  | **DEGs** | **DCPs** | **DEGs Correlation network** |
| --- | --- | --- | --- |
| Missing allowed | 30% max (BRB) | 30% max | Inherited from DEGs |
| Mean p-value (BRB) | 0.05 |  | Inherited from DEGs |
| Regulation | same in all studies |  | Inherited from DEGs |
| Correlation p-value |  | if p-value > 0.2, marked as NOT significantly correlated | < 0.2 |
| Correlation direction |  | same in all studies present for at least one state | Same in all studies, for each separate state |
| Difference of correlation p-value |  | < 0.1 |  |
| Minimum number of studies present | 2 out of 2 | 2 out of 2 | Inherited from DEGs |
| Sample size |  | > 2 |  |
| Difference of correlation direction |  | 2 out of 2 |  |
| FDR on Fisher p-value | < 0.1 | < 0.02 | < 0.025 |
| PUC |  |  | Applied after FDR filter |
| Procedure for  duplicate Gene symbol |  | Select pair with lower difference of correlation fisher p-value.  Remove daps that have the same gene symbol combination but different probe ids and have different change of correlation direction | If Different probe IDs have the same Gene symbol, they are going to be interpreted as the same gene in the network. |

Supplementary Table S4: All filters for all calculations on cervical cancer data

|  | **DCPs** | **DEGs Correlation network** | **DEGs Local Partial Correlation** |
| --- | --- | --- | --- |
| Missing allowed | 30% max |  | 30% max in separate states |
| Correlation p-value | if pv > 0.2, marked as NOT significantly correlated | < 0.1 | Significant in DEGs Correlation network |
| Correlation direction | same in all studies present for at least one state | Same in all studies, for each separate state |  |
| Local Partial correlation p-value |  |  | < 0.4 |
| Local Partial correlation direction |  |  | Same in all studies, for each separate state |
| Difference of correlation p-value | < 0.1 |  |  |
| Minimum number of studies present | 3 out of 5 |  |  |
| Sample size | > 2 |  |  |
| Difference of correlation direction | same in all studies present |  |  |
| FDR on Fisher p-value | < 0.0025 | < 10^-8^ | < 0.05 |
| PUC |  | Applied after FDR filter |  |
| Procedure for  duplicate Gene symbol | Select pair with lower difference of correlation fisher pv. | Select pair with lower correlation fisher pv. (Nothing done when they show different directions in correlation) | Select pair with higher correlation. (Nothing done when they show different directions in correlation) |

Differentially Co-expressed Pairs/Genes (DCPs/DC genes).

For both biological systems studied in this paper we identified the Differentially Co-expressed Pairs using the same procedure. We start considering all genes in the dataset and filter out the genes presenting more than 30% missing data. Next, we calculate for each possible pair of genes their correlation in 2 different states and then the difference of correlation between those states and filtered out pairs that are not present in at least a fixed number of datasets (BcKO: 2 (all studies), cervical cancer: 3 out of 5) and do not have sample size greater than 2. In all datasets the difference between correlations in two states must have the same direction (sign). To assure similarities between datasets we select the pairs that have the same direction (sign) of correlation at a significance level of 20% in at least one state. This way we ascertain that the pair is correlated in at least one state and has the same behavior in the state where the correlation occurs. We then proceed to the computation of the p-value for the difference of correlation [21] and only keep the pairs with p-value lower than 20% in all studies. Now meta-analysis is done through Fisher’s method and then FDR. Next we eliminate the pairs that show FDR higher than a threshold (Table S3, S4). The final step is to identify the pairs that passed the FDR filter and were considered significantly correlated in the final reconstructed network (correlation network for BcKO and local partial correlation network for cervical cancer). Differentially Co-expressed Genes for BcKO and cervical cancer can be found in Dataset 3 and Table 1 (DCPs – cancer) respectively.

Bi-Partite Betweeness Centrality

**Bi-Partite Betweeness Centrality points to possible bottlenecks.**

Figure S1. Hypothetic example of causal genes (nodes in red) communicating with all genes through DCPs (edges in black).

**Permutation analysis**

To confirm that these high values of Bi-Partite Betweenness Centrality are not random we performed a permutation analysis. It was done in the following way:

1. Calculate the difference between the mean of bipartite betweenness centrality for DCP genes and for the rest of the genes. We will call it the reference value.
2. Randomly select 14 genes out of all genes (permutation) and calculate the difference between the mean of bipartite betweenness centrality for the randomly selected genes and for the rest of the genes.
3. Repeat step 2 ten thousand times.
4. Plot the histogram of the mean differences from step 3 along with the reference value (step 1)
5. Repeat Steps 1-4 for median, first and third quartiles.

If the reference value for a parameter is located to the right of the histogram then we have evidence that this parameter is higher for DCP genes then for a random sample from the population of bi-partite betweenness centrality values. If this happens for all graphs, then we can say that DCP genes present values of bi-partite betweenness centrality entirely concentrated in higher values then the rest of the genes. Figure S5 illustrates our results for cervical cancer datasets.


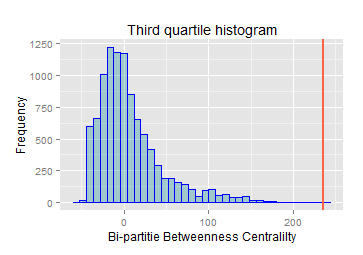

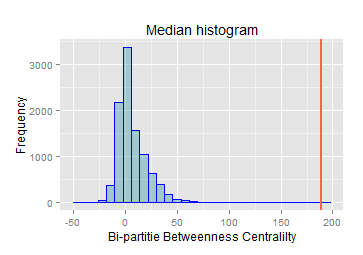

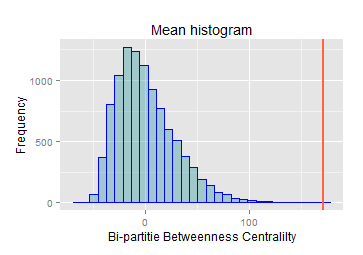

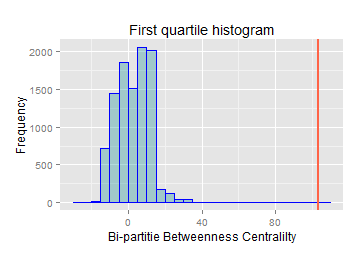


Figure S2. Figures showing the histogram of the difference between the values of a parameter (mean, median. 1st quartile and 3^rd^ quartile) from permutation samples of betweenness centrality to the value of the same parameter in the population betweenness centrality data. Note that in all histograms the difference of a parameter of betweenness centrality between the original DCG sample and the population (red line) is situated extremely to the right which shows us that the betweenness centrality amongst only DCG are concentrated in higher values compared to the other genes.


Scripts

Local Partial Correlation (R script for calculation)

suppressMessages(library("parallel"))

suppressMessages(library("foreach"))

suppressMessages(library("doParallel"))

### Functions ###

filter.rows <- function(x, rows, fun=sd){

rows <- min(rows, nrow(x))

val <- apply(x, 1, fun)

sel.rows <- order(val, decreasing=TRUE)[1:rows]

return(x[sel.rows, ])}

just.filter <- function(x, percent, maxRows){

res <- filter.rows(filter.prop(x, percent), maxRows)

return(res)}

# Calculate Pearson correlation (upper triangle) and its p-value (lower triangle).

cor.prob = function(X, dfr = nrow(X) - 2) {

R = cor(X)

above = row(R) < col(R)

r2 = R[above]^2

Fstat = r2 * dfr / (1 - r2)

#Probability of the F distribution

R[above] = 1 - pf(Fstat, 1, dfr)

R}

# alpha=0.01, pvalue threshold for correlation.

#It defines the starting adjacency matrix matRoXY.

calcLPC <- function(data, nLim, porc=.7, alpha=0.01, save=TRUE, numOfCores=NA) {

print(paste("alpha =", alpha))

nCores = detectCores() - 1

cl = makeCluster(nCores)

registerDoParallel(cl, cores = detectCores() - 1)

sampleNames <- toupper(names(data))

sampleNames <- gsub("[_,.,;,-,,|,/,\\].*", "", sampleNames)

names(data) <- sampleNames

if (nLim != -1) {

data <- just.filter(data, porc, nLim)

print("filtering")

} else {

print("NOT filtering")

}

dim(data)

#Gene expression data (p x n), p variables and n samples

#X=as.matrix(read.table(file = "your_data.txt", sep = "", stringsAsFactors=F))

X=t(data)

p = ncol(X)

n = nrow(X)

#Warning for the case n >= p.

if(p <= n){

print("nothing to do: n > p.")

return(FALSE)

}

#Calculate the sample covariance

covX = cov(X)

#Calculates the sample correlation

corX = cor(X)

pvalcor = cor.prob(X)

# New empty matrix

matRoXY = matrix(0, ncol = p, nrow = p)

colnames(matRoXY) = colnames(X)

#Fill the matrix matRoXY with correlation from X

# If alpha = 1, preserve all values of correlationa,

# else, preserve values of correlation which p-value < alpha.

if (alpha==1) {

for(i in 1:(p-1)) {

for(j in (i+1):p) {

matRoXY[i,j] = corX[i,j]

matRoXY[j,i] = matRoXY[i,j]

}

}

} else {

for(i in 1:(p-1)) {

for(j in (i+1):p) {

matRoXY[i,j] = ifelse(pvalcor[i,j] < alpha, corX[i,j], 0)

matRoXY[j,i] = matRoXY[i,j]

}

}

}

#Fills with zeros the NA entries.

matRoXY[is.na(matRoXY)] = 0

# igraph function (creates an igraph object from the adjacency matrix)

g1 = graph.adjacency(matRoXY, mode = ("undirected"), weighted = TRUE)

#Set the vertices names to the sequence 1:p.

V(g1)$name = c(seq(1:p))

#Accessing edges.

edges = get.adjlist(g1)

totEdg = unlist(edges)

#Generate a matrix with 99 in all positions (not to get confused with p-values)

Ap = matrix(99,ncol=ncol(X),nrow=ncol(X))

#Maximum number of neighbors is equal to the sample size.

nViz = n

t0 = Sys.time()

lpcData = foreach(k = 1:ncol(X), .packages = c("igraph", "corpcor"), .combine = rbind) %dopar% {

#Get values from the list "edges"

#Position where the elements is different from zero.

vertices = edges[[k]]

'print(k)

print(vertices)

print(length(vertices))

print("-----------")'

#Will pass to the line of the matrix

if (length(vertices) > 0) {

for (j in 1:length(vertices))

{

if(vertices[j] > k && matRoXY[k, vertices[j]]!=0)

{

i1 = as.character(k)

j1 = as.character(vertices[j])

#Get the neighbors of a pairo of nodes.

vizlist = graph.neighborhood(g1, 1, nodes = c(i1, j1), mode=c("all"))

viz = sort(as.integer(unique(c(V(vizlist[[1]])$name, V(vizlist[[2]])$name)),decreasing=TRUE))

# Downsizing the number of neighbors in case we have we still

# have more neighbors than observations.

if(length(viz) >= nViz)

{

#Get the biggest absolute value of correlation from the two

#selected nodes.

cor.maiores.i = sort(abs(matRoXY[viz, c(k)][-k]), decreasing = TRUE)[1:(nViz/2)]

cor.maiores.j = sort(abs(matRoXY[viz, c(vertices[j])][-(vertices[j])]),

decreasing = TRUE)[1:(nViz/2)]

#Remove the zeros

cor.maiores.i = cor.maiores.i[cor.maiores.i > 0]

cor.maiores.j = cor.maiores.j[cor.maiores.j > 0]

#Selecting n/2 vertices with highest correlations

cor.maiores = sort(abs(unique(c(cor.maiores.j,cor.maiores.i))), decreasing =

TRUE)[1:(nViz/2)]

cor.maiores.i = cor.maiores.i[cor.maiores.i %in% cor.maiores]

cor.maiores.j = cor.maiores.j[cor.maiores.j %in% cor.maiores]

majI = cor.maiores.i[! is.na(cor.maiores.i)]

majJ = cor.maiores.j[! is.na(cor.maiores.j)]

posicao.i = sapply(majI, function(valmajI){which(abs(matRoXY[ ,c(k)]) == valmajI,

useNames = TRUE)})

posicao.i = unlist(posicao.i)

posicao.j = sapply(majJ, function(valmajJ){which(abs(matRoXY[ ,vertices[j]]) ==

valmajJ, useNames = TRUE)})

posicao.j = unlist(posicao.j)

if((length(posicao.i)==0) & (length(posicao.j)==0)) next

if(length(posicao.j)==0){

viz = sort(c(k, as.numeric(vertices[j]) , posicao.i))

} else {

if (length(posicao.i)==0) viz <- sort(c(k, as.numeric(vertices[j]) , posicao.j))

else viz = unique(sort(c(k, as.numeric(vertices[j]), c(posicao.i, posicao.j))))

}

}

lcl = length(viz)

#Selecting neighbor-and-pair covariance matrix

Csub = covX[viz,viz]

# Calcultaes the partial correlation from

# neighbor-and-pair covariance matrix

corr.inv = cor2pcor(Csub)

rownames(corr.inv) = viz

colnames(corr.inv) = viz

Csub2 = Csub

dfr = n-2

R = corr.inv

above = row(Csub2) < col(Csub2)

r2 = R[above]^2

Fstat = r2 * dfr / (1 - r2)

R[above] = 1 - pf(Fstat, 1, dfr)

# Upper triangle lpcData = partial correaltion

# Lower triangle lpcData = p-value

Ap[k,vertices[j]] = R[i1,j1]

Ap[vertices[j],k] = R[j1,i1]

}

} # loop

}

Ap[k,]

}

ti <- Sys.time()

print(paste( as.numeric( round(ti-t0,2), units = "secs")/60, "min, p =", p, "tempo/p=", round(as.numeric(ti-t0, units = "secs")/p,2), "s/node"))

colnames(lpcData) = rownames(data)

rownames(lpcData) = rownames(data)

if (save) {

writeLpcFile(lpcData, porc, pGenes=p)

}

stopCluster(cl)

return(lpcData)

}
