# Supplementary material for: Differentially correlated genes in co-expression networks control phenotype transitions
Source: F1000Res. 2016 Nov 22;5:2740. [Version 1] doi: 10.12688/f1000research.9708.1 (PMC5247791; doi:10.12688/f1000research.9708.1)
Supplement: Peer review history

# Peer review: Referee response for version 1

**DOI:** 10.5256/f1000research.10464.r17875

**Published:** 2017-01-16

**Authors:** Fabrício Martins Lopes

**Affiliations:** 1 Federal University of Technology – Paraná, Cornélio Procópio, Brazil

The manuscript “Differentially correlated genes in co-expression networks control phenotype transitions” investigates the differentially co-expressed genes in two biological processes, a homogeneous one-causal-factor process (B cell deficiency) and a heterogeneous multi-causal system (cervical cancer). The authors have adopted the Pearson correlation and partial correlation for the inference of networks.

**Major revision:**

- The networks were inferred from local partial correlation method, which is able to identify a linear relationship between two variables X and Y (genes), and this relationship may or may not be mediate by another gene Z. It is not clear why the authors have adopted the Pearson correlation for B cell deficiency analysis and the partial correlation for cervical cancer analysis. Moreover, it would be interesting to highlight the gain obtained by adopting the partial correlation. For instance, what were the relationships inferred with the partial correlation that would not be inferred using Pearson correlation?
- Another important issue is that even with partial correlation, only pairwise of relationships are identified. In the study presented at http://dx.doi.org/10.1109/JSTSP.2008.923841, it presents the Intrinsically Multivariate Predictive (IMP) Genes, which are genes that depend on a subset of predictors. How did the authors deal with these IMP genes?
- It is not clear how and why the microarray data was filtered. The authors could better describe how the data was filtered and how the parameters were adopted.
- The title “Differentially correlated genes in co-expression networks control phenotype transitions” is too rigid leading to the understanding that all correlated genes control the phenotype transitions. I believe that is not true. Authors could provide a more appropriate title.

**Minor revisions:**

- Page 3: “homogenous” → homogeneous
- Page 4: "correlation lost" → correlation loss;

I have read this submission. I believe that I have an appropriate level of expertise to confirm that it is of an acceptable scientific standard, however I have significant reservations, as outlined above.

## References

[1] MartinsDBraga-NetoUHashimotoRBittnerMDoughertyE: Intrinsically Multivariate Predictive Genes. *IEEE Journal of Selected Topics in Signal Processing*.2008;2(3) : 10.1109/JSTSP.2008.923841 424-439 10.1109/JSTSP.2008.923841

# Peer review: Referee response for version 1

**DOI:** 10.5256/f1000research.10464.r17872

**Published:** 2016-12-23

**Authors:** Andrei Zinovyev

**Affiliations:** 1 Institut Curie, Paris, France

The manuscript "Differentially correlated genes in co-expression networks control phenotype transitions" by Lina Thomas *et al*, is devoted to describing a case study of two transcriptomic datasets with the focus on characterizing pairs of differentially correlated genes, with a limited experimental validation of the conclusions of the statistical analysis.

 The manuscript is clear, technically sound and exploits an interesting approach for the analysis of expression data. The conclusions of the statistical analysis are sufficiently justified. I like the detailed and illustrated description of the novel methods exploited the paper. Experimental validation of several findings is a big plus. Therefore, I think the article deserves to be indexed.

 I have several remarks for the manuscript which I think should be addressed before approval:

1. I am not completely comfortable with the title of the manuscript, which is quite conceptual, while the content of the paper remains descriptive and does not provide mechanistic insight on how DCG pairs can control the phenotype. I suggest to the authors to have some reflexion on how to make it more adequate.
2. Related to 1), in Introduction the desciption of the mechanisms by which DCG pairs can "contribute to propagation of perturbation" remain very illusive. I suggest to the authors to formulate more clearly at least several hypotheses or scenario by which DCG pairs might appear and play an important role. A figure illustrating such hypotheses would clarify what the authors mean.
3. Figures 1 and 3 are not very informative. Can authors make an effort to improve this aspect (at least, visualize some DC gene names?)
4. The authors do not discuss a possibility that appearance of DC pairs can be a result of differential sample tissue composition from several cell types (i.e., immune cells in jejunum or in a tumor tissue). Discussing this point would be an advantage.
5. In several places, the authors apply terms "upstream", "downstream" with respect to the network which is undirected by its nature. I suggest to underline that the nature of correlation networks does not allow distinguishing causality direction and considering genes "downstream" of the key drivers is only a hypothesis which can not be assessed from the data.
6. Description of the transcriptomic datasets in Materials and Methods is too brief, especially for the cervix dataset which seems to be quite composite. It would be appropriate to specify more clearly the dataset's composition (not simply referring to the original publications) directly in the paper text.
7. One thing which is confusing to me is that the correlation networks are constructed differently for two case studies (direct vs partial). I understood the reason why partial correlation was prefered for the cervical cancer study, however, the question is: can the conclusion about that DC pairs do not contain key drivers in the case of cervical cancer be affected by the difference in the methodology of correlation graph computation? It would be usefull to clarify this aspect.
8. The section "Filtering and meta-analysis of microarray data" was not clear to me. I suggest to re-write it.

Minor remarks:

 Page 4: "correlation lost" -> "correlation loss"

 Description of the partial correlation method refers to a paper (30) which can not be easily accessed. Direct reference to the arXiv preprint would be more appropriate in this case

I have read this submission. I believe that I have an appropriate level of expertise to confirm that it is of an acceptable scientific standard, however I have significant reservations, as outlined above.

# Peer review: Referee response for version 1

**DOI:** 10.5256/f1000research.10464.r17874

**Published:** 2016-12-20

**Authors:** Thiago M. Venancio

**Affiliations:** 1 Center for Bioscience and Biotechnology, State University of Norte Fluminense Darcy Ribeiro, Campos dos Goytacazes, RJ, 28035-200, Brazil

In the present work Thomas *et al. *employed method to find differentially co-expressed genes in co-expression networks of a B lymphocyte deficiency (largely mono-causal) and a complex cervical cancer (multi-causal) dataset. They used different graph-theoretical approaches to find relevant genes in this context.

 Interestingly, the authors found that 72% (39/54) of the correlation gains involve at least one Ig gene, which is in agreement with the previously shown association between intestinal gene expression and B cells ability to produce antibodies. Is it possible that this "correlation gains" are merely a consequence of the general enrichment of Ig genes in the DC list?

 In the cervical cancer analysis, the authors used shortest-path and betweenness centrality to argue for the regulatory relevance of DC genes. I think it would be great to supplement this finding with more biochemical information. For example, how many of these genes are transcription factors or protein kinases?

 Overall, I think this study is technically sound and properly executed.

 == Minor corrections

 In the abstract, "mus musculus" should read "Mus musculus".

 In Figure 2, Ig is underlined as if marked by a spellchecker.

I have read this submission. I believe that I have an appropriate level of expertise to confirm that it is of an acceptable scientific standard.
